# Prevalence and factors associated with caesarean section in Rwanda: a trend analysis of Rwanda demographic and health survey 2000 to 2019–20

**DOI:** 10.1186/s12884-022-04679-y

**Published:** 2022-05-16

**Authors:** Peter M. Kibe, Grace Wambura Mbuthia, Duncan N. Shikuku, Catherine Akoth, James Odhiambo Oguta, Loise Ng’ang’a, Samwel Maina Gatimu

**Affiliations:** 1 African Population and Health Research Centre, Nairobi, Kenya; 2 Department of Community Health, Jomo Kenyatta University of Agriculture and Technology, Juja, Kenya; 3 Liverpool School of Tropical Medicine, Liverpool, UK; 4 Institute of Tropical and Infectious Diseases, University of Nairobi, Nairobi, Kenya; 5 School of Health and Related Research, University of Sheffield, Sheffield, UK; 6 Health Section, UNICEF, Eastern and Southern Africa Regional Office, Nairobi, Kenya; 7 Partners in Health, Kigali, Rwanda; 8 Department of Economics, Population and Development Studies, University of Nairobi, Nairobi, Kenya; 9 Diabetic Foot Foundation Kenya, Nairobi, Kenya

**Keywords:** Rwanda, Caesarean section, Women, Maternal health, Maternal mortality, Eastern Africa

## Abstract

**Background:**

Caesarean section (CS) is an important medical intervention for reducing the risk of poor perinatal outcomes. However, CS trends in sub-Saharan Africa (SSA) continue to increase yet maternal and neonatal mortality and morbidity remain high. Rwanda, like many other countries in SSA, has shown an increasing trend in the use of CS. This study assessed the trends and factors associated with CS delivery in Rwanda over the past two decades.

**Methods:**

We used nationally representative child datasets from the Rwanda Demographic and Health Survey 2000 to 2019–20. All births in the preceding 3 years to the survey were assessed for the mode of delivery. The participants’ characteristics, trends and the prevalence of CS were analysed using frequencies and percentages. Unadjusted and adjusted logistic regression analyses were used to assess the factors associated with population and hospital-based CS in Rwanda for each of the surveys.

**Results:**

The population-based rate of CS in Rwanda significantly increased from 2.2% (95% CI 1.8–2.6) in 2000 to 15.6% (95% CI 13.9–16.5) in 2019–20. Despite increasing in all health facilities over time, the rate of CS was about four times higher in private (60.6%) compared to public health facilities (15.4%) in 2019–20. The rates and odds of CS were disproportionately high among women of high socioeconomic groups, those who resided in Kigali city, had multiple pregnancies, and attended at least four antenatal care visits while the odds of CS were significantly lower among multiparous women and those who had female babies.

**Conclusion:**

Over the past two decades, the rate of CS use in Rwanda increased significantly at health facility and population level with high regional and socio-economic disparities. There is a need to examine the disparities in CS trends and developing tailored policy guidelines to ensure proper use of CS in Rwanda.

**Supplementary Information:**

The online version contains supplementary material available at 10.1186/s12884-022-04679-y.

## Background

The burden for maternal and neonatal mortality remains disproportionately high in many low- and middle-income countries (LMICs) [1, 2]. About 99% of all global maternal deaths occurred in LMICs between 1990 and 2010 with an estimated two-thirds occurring in sub-Saharan Africa (SSA) [1, 2]. Despite the high maternal deaths in SSA, Rwanda recorded a 79% decline in maternal mortality ratio between 2000 and 2017 [1]. This reduction has been accelerated by increased access and use of skilled and competent care by women during the pregnancy continuum. However, about 73% of the global maternal deaths remain due to direct obstetric causes including obstetric interventions and complications [3] despite most of them being preventable. The provision of basic and comprehensive emergency obstetric and newborn care (EmONC) within a continuum of care is essential to reduce maternal and newborn morbidity and mortality [4, 5].

Caesarean section (CS) is an important comprehensive EmONC signal function and medical intervention for reducing risks of perinatal morbidity and mortality [6]. The proportion of delivery by CS is used to estimate the level of access and use of the intervention in saving maternal and child health at a population level [7]. Over the past few decades, global trends have shown a steady increase in CS use [8]. Gibbons and colleagues found that half of the countries surveyed globally had CS rates of above 15% [9] while Betran and colleagues estimated that by 2030, 29% of women will be giving birth through CS globally; about 7% in SSA and 63% in Eastern Asia [10]. Countries in SSA are also reporting high CS rates despite the high burden of preventable maternal and neonatal mortality [1]. However, huge disparities in CS use exist in SSA countries with some countries like South Sudan reporting rates lower than 1% while others like Ghana reporting a prevalence of 16% [11, 12].

These observed global increases in CS are driven majorly by medical and non-medical factors [6, 9]. Medically, CS deliveries are indicated in high risk conditions such as placenta previa, breech presentation, contracted pelvis, post-term pregnancy and hypertensive disorders [13]. However, CS deliveries have been conducted due to non-medical factors including maternal age, socioeconomic status, literacy levels, occupation, religion and culture [12, 14] and other demand-driven factors such as private practice and the cost of accessing CS [12]. In LMICs, the causes for the increasing CS use remain unclear though socioeconomic factors have been shown to contribute to the increase [6, 15]. The World Health Organization (WHO) recommends an optimal CS rate of 10–15% where rates below the lower limit suggest the unmet need for CS while rates above the upper limit suggest overuse of CS [16, 17]. The use of CS without medical indication is of no significance in reducing maternal and child mortality [6, 7], with recent evidence showing no beneficial effect of CS rates of above 10% on perinatal mortality rate [7].

Although Rwanda has made great strides in reducing maternal and child mortality in recent years, there is a risk of reversing these gains through the overuse of CS which has been associated with increased risk of adverse outcomes especially for constrained health systems in many LMICs [18, 19]. Rwanda, like many countries in SSA, has shown a consistent rise in the use of CS, which is estimated to account for between 13% [20] to 25.9% [21] of all deliveries. A recent study reported CS rates of 64% in private health facilities in Rwanda [11]. In addition, evidence indicates that if uncontrolled, the use of CS will continue to rise and with it the risk of adverse consequences [20]. Therefore, understanding the trends and patterns of CS use is the mainstay of preventing risks and developing context-specific evidence for policies to reduce maternal and neonatal mortality. However, comprehensive evidence on trends and patterns of CS use is lacking in Rwanda. This study used national-level data from the Rwanda Demographic and Health Survey between 2000 and 2019–20 to assess the trends and factors associated with CS delivery in Rwanda at both population and hospital levels.

## Methods

### Study setting

Rwanda is a low-income, agricultural and landlocked country with approximately 11 million people living in five regions covering an area of 26,338 km^2^ [21]. It has an average of 4.4 persons per household [22] and a gross domestic product per capita of US $780.80 [23]. About half (48%) of its population is under 19 years of age and 39% live below the poverty line with a life expectancy at birth of 71.1 years for women and an adult literacy rate of 80% among 15–49 years old women. In addition, 87.3% of the population has health insurance and access to health services; spending an average of 47.4 min to reach a health centre [21]. In 2016, CS in Rwanda were conducted in 27 (75%) of the 36 districts, provincial and referral hospitals [21].

### Data source and sample

The study used the child datasets from the Rwanda Demographic Health Surveys (RDHS) conducted in 2000, 2005, 2010, 2014–15 and 2019–20 using stratified, two-stage cluster sampling [22, 24–27]. Households were stratified into urban or rural and all eligible women 15–49 years in selected households were interviewed using standard DHS questionnaires. All babies born within the preceding 3 years of each survey and with complete data were included in the population-based analysis while only babies delivered at a hospital were included in the hospital-based analysis. Of the 75,777 children born within the 3 years preceding each survey, 34,144 children were included after excluding 41,633 children with missing observations in the outcome variable as explained by the guide to DHS statistics [28] (Suppl. Fig. 1).

### Measures

**Fig. 1:**
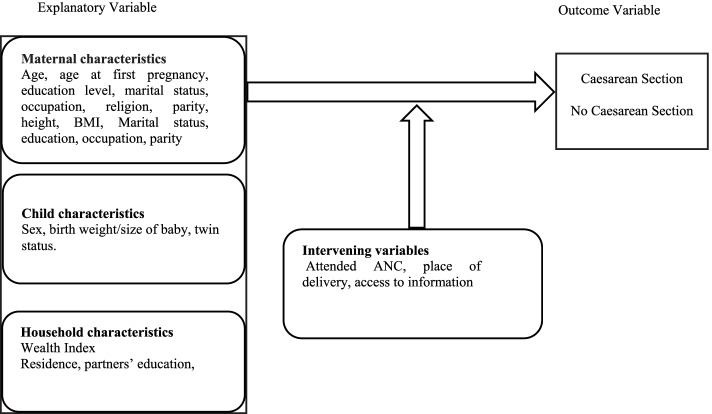
Conceptual framework on factors associated with CS use (adapted from Kizito 2021)

We conceptualized our study variables using the framework adapted from Kizito and Schuemacher [29] as shown in Fig. 1. The outcome variable was delivery by CS, which was categorized into “Yes” or “No”. Women were asked if they had been delivered by CS within the 3 years preceding the survey. Since it is possible for women to have more than one CS in 3 years, we used the participants’ unique identifiers and weighted samples to account for the clustering of CS. The explanatory variables included in the study were identified from a review of literature on factors associated with CS use [12, 14, 30–35]. Supplementary Table 1 operationalises these variables. The explanatory variables were categorised into maternal, child and household characteristics. Intervening variables were a set of variables acting on the explanatory variables and included access to information (Yes or No), place of delivery (private, public and home/others) and antenatal care (ANC) attendance (< 4 and ≥ 4 visits and missing). Maternal characteristics included residence (urban or rural), maternal age at birth (< 20, 20–34 and ≥ 35 years), education status (no formal, primary and secondary or higher), marital status (in-a-union and not-in-a-union), occupation (not working, agricultural and formal employment), parity (1, 2–4, ≥5) and region (East, West, South, North and Kigali City). Child characteristics included the weight of the baby (normal [2500-4000 g], low birth weight [< 2500 g] and big baby [> 4000 g]), low birth weight and big baby) [36], sex of the baby (male or female) and type of pregnancy (singleton or multiple). Household characteristics included household income and partner’s education (no formal, primary and secondary or higher).

### Statistical analysis

We performed analyses of datasets using Stata version 17.0 (Stata Corporation, College Station, TX). We applied design based analysis using DHS sample weights and adjusted for sample errors using svy command. Participants’ characteristics and trends in the prevalence of CS were analysed using frequencies and percentages. To assess the factors associated with population-based and hospital-based CS, bivariate and multivariable logistic regression models were performed for each of the surveys. Variance inflation factor was used to assess multicollinearity. All variables included in the unadjusted model were hierarchically included in the adjusted model due to their importance in explaining CS and guided by the conceptual framework by Kizito and Schuemacher [29]. We reported both the unadjusted and adjusted odds ratios and considered significance at a *p*-value of less than or equal to 0.05. Only children with complete data were included in the analyses. The reporting in this study were guided by the STROBE guidelines for cross-sectional studies (Suppl. Table 3) [37].

### Ethics

The study used anonymised open-access secondary data from the RDHS, which received ethical approvals from the Rwanda National Ethics Committee and the Institutional Review Board of ICF International. The data were accessed upon approval of data request to the DHS program and were used as per the data agreement. Additional information on the ethical approvals and processes for the surveys can be obtained from the published reports [21, 23–25].

## Results

### Respondents’ characteristics

Overall, the study included 34,144 children born within the 3 years preceding each survey (Suppl. Fig. 1). A majority of the children were born to middle-aged women (20–34 years), women who resided in rural areas, had a primary level of education, had two to four children, and had attended less than four ANC visits across the years. An almost equal proportion of participants lived in the West, East, and South provinces of Rwanda with the smallest proportion of participants living in Kigali City (Table 1).

**Table 1:** Participants’ characteristics

Characteristics	2000 (***N*** = 6706)		2005 (***N*** = 7712)		2010 (***N*** = 7159)		2014–15 (***N*** = 6248)		2019–20 (***N*** = 6319)	
	n	%	n	%	n	%	n	%	n	%
**Age, years**										
15–19	431	6.4	318	4.1	342	4.8	344	5.5	286	4.5
20–34	4881	72.8	5830	75.6	5605	78.3	4875	78.0	4520	71.5
35–49	1394	20.8	1564	20.3	1212	16.9	1029	16.5	1513	23.9
**Residence**										
Urban	1460	21.8	1478	19.2	906	12.7	1384	22.2	1284	20.3
Rural	5246	78.2	6234	80.8	6253	87.3	4864	77.9	5035	79.7
**Marital status**										
Not in a union	841	12.5	904	11.7	988	13.8	1032	16.5	1004	15.9
Married/cohabiting	5865	87.5	6808	88.3	6171	86.2	5216	83.5	5315	84.1
**Education**										
No formal	2115	31.5	2082	27.0	1327	18.5	893	14.3	691	10.9
Primary	3802	56.7	4941	64.1	5222	72.9	4479	71.7	4076	64.5
Secondary and higher	789	11.8	689	8.9	610	8.5	876	14.0	1552	24.6
**Occupation**										
Not working	149	2.6	1552	20.1	831	11.6	534	8.6	1269	20.1
Agriculture	4915	86.4	5475	71.0	5473	76.5	4572	73.2	2510	39.7
Formal employment	624	11.0	685	8.9	855	11.9	1140	18.3	2540	40.2
**Wealth, quintiles**										
Poorest	1191	18.0	1604	20.8	1703	23.8	1542	24.7	1604	25.4
Poorer	1587	24.0	1518	19.7	1567	21.9	1294	20.7	1241	19.6
Average	824	12.5	1517	19.7	1419	19.8	1152	18.4	1178	18.6
Richer	1300	19.7	1591	20.6	1289	18.0	1067	17.1	1142	18.1
Richest	1707	25.8	1482	19.2	1181	16.5	1193	19.1	1154	18.3
**ANC attendance, visits**										
< 4	3452	51.5	50.85	50.0	2929	40.9	39.36	38.1	2367	37.5
≥4	442	6.6	7.659	7.0	1668	23.3	31.33	29.9	2102	33.3
Missing	2812	41.9	41.5	40.8	2562	35.8	29.31	28.4	1850	29.3
**Parity**										
1	694	10.4	786	10.2	1173	16.4	1222	19.6	1141	18.1
2–4	3505	52.3	3925	50.9	3776	52.7	3617	57.9	3612	57.2
5+	2507	37.4	3001	38.9	2210	30.9	1409	22.6	1566	24.8
**Baby’s sex**										
Male	3415	50.9	3923	50.9	3601	50.3	3149	50.4	3205	50.7
Female	3291	49.1	3789	49.1	3558	49.7	3099	49.6	3114	49.3
**Baby’s birth weight**										
Average	1591	23.8	1855	24.1	3844	53.7	4581	73.4	4839	76.6
Low birth weight	218	3.3	207	2.7	501	7.0	600	9.6	692	11.0
Big baby	4872	72.9	5650	73.3	2814	39.3	1061	17.0	788	12.5
**Twin Status**										
Singleton	6542	97.6	7518	97.5	6959	97.2	6069	97.1	6151	97.3
Multiple	164	2.5	194	2.5	200	2.8	179	2.9	168	2.7
**Access to information**										
No	2375	35.4	1661	21.5	673	9.4	1028	16.5	1359	21.5
Yes	4331	64.6	6051	78.5	6486	90.6	5220	83.6	4960	78.5
**Partner’s education**										
No formal	1916	29.8	1986	27.1	1318	19.7	906	15.9	673	12.7
Primary	3551	55.2	4409	60.1	4649	69.6	4049	71.2	3521	66.5
Secondary and higher	961	15.0	945	12.9	715	10.7	730	12.8	1103	20.8
**Region**										
Kigali City	837	12.5	504	6.5	750	10.5	729	11.7	731	11.6
West	1605	23.9	1906	24.7	1805	25.2	1624	26.0	1681	26.6
East	1533	22.9	1973	25.6	1790	25.0	1545	24.7	1512	23.9
South	1490	22.2	2014	26.1	1771	24.7	1503	24.1	1432	22.7
North	1241	18.5	1315	17.1	1043	14.6	847	13.6	963	23.9
**Place of delivery**										
Home/others	4723	70.5	5429	70.5	2005	28.0	525	8.4	435	6.9
Public health facility	1856	27.7	2169	28.2	5101	71.3	5702	91.3	5775	91.4
Private health facility	121	1.8	98	1.3	50	0.7	18	0.3	109	1.7

### Trends in the prevalence of CS in Rwanda

**Fig. 2:**
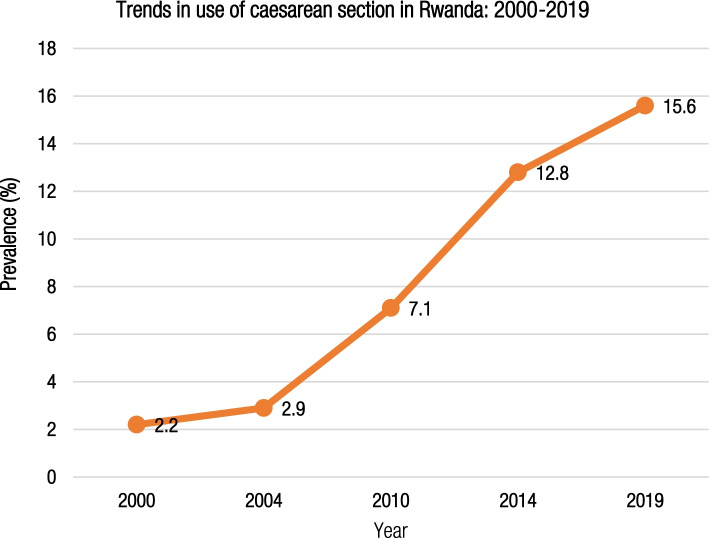
Trends in use of CS in Rwanda from the year 2000 to 2019–20

Overall, the rate of CS increased significantly from 2.2% (95% CI 1.8–2.6) in 2000 to 15.6% (95% CI 13.9–16.5) in 2019–20 (Fig. 2). The rate was consistently high among women 15–19 years of age, women residing in urban areas, those with secondary or higher levels of education, from the richest households, with access to information, on paid employment, and those who had one child, male babies, and multiple pregnancies across the years (Table 2).

**Table 2:** Prevalence of caesarean section according to participants’ characteristics

Characteristics	2000		2005		2010		2014–15		2019–20	
	%	95% CI	%	95% CI	%	95% CI	%	95% CI	%	95% CI
**Age, years**										
15–19	3.7	(2.0–6.9)	4.1	(2.3–7.2)	9.9	(7.0–13.8)	14.1	(10.3–18.9)	14.2	(10.1–19.6)
20–34	2.2	(1.8–2.8)	3.1	(2.5–3.7)	7.4	(6.5–8.3)	13.3	(12.1–14.7)	15.8	(14.3–17.4)
35–49	1.5	(0.9–2.5)	1.8	(1.2–2.6)	5.2	(3.9–6.9)	9.7	(7.8–12.0)	13.5	(11.2–16.1)
**Residence**										
Urban	6.4	(5.0–8.1)	7.4	(5.5–9.97)	16.0	(13.0–19.6)	22.1	(18.9–25.8)	14.7	(12.4–17.4)
Rural	1.4	(1.1–1.9)	2.1	(1.7–2.6)	6.0	(5.3–6.8)	10.8	(9.7–12.1)	15.3	(13.9–16.7)
**Marital status**										
Not in a union	1.6	(1.0–2.7)	3.9	(2.6–5.7)	7.8	(6.2–9.7)	13.1	(10.9–15.7)	26.4	(22.9–30.3)
Married/cohabiting	2.2	(1.8–2.7)	2.7	(2.2–3.3)	7.0	(6.2–7.9)	12.7	(11.5–14.1)	12.9	(11.6–14.3)
**Education**										
No formal	1.1	(0.7–1.7)	2.2	(1.5–3.1)	5.1	(3.9–6.8)	7.7	(5.7–10.3)	8.9	(6.7–11.9)
Primary	2.3	(1.8–3.0)	2.4	(1.9–3.1)	6.4	(5.6–7.2)	12.1	(10.8–13.5)	12.7	(11.4–14.2)
Secondary	5.1	(3.7–7.0)	8.4	(6.1–11.5)	18.6	(15.0–22.8)	22.4	(19.1–26.1)	24.3	(21.1–27.9)
**Occupation**										
Not working	7.7	(3.2–17.1)	4.6	(3.4–6.2)	9.4	(7.4–11.9)	13.9	(11.2–17.2)	19.0	(16.4–22.0)
Agriculture	1.4	(1.0–1.8)	1.9	(1.5–2.4)	5.7	(5.0–6.6)	10.0	(8.9–11.2)	12.2	(10.5–14.0)
Formal employment	6.1	(4.1–8.9)	7.2	(5.1–10.1)	14.2	(11.4–17.6)	24.5	(20.9–28.5)	16.2	(14.3–18.3)
**Wealth, quintiles**										
Poorest	1.6	(0.9–2.8)	1.0	(13.7–18.0)	5.5	(4.5–6.8)	9.7	(7.9–11.8)	10.4	(8.6–12.6)
Poorer	1.0	(0.6–1.8)	2.1	(1.3–3.3)	5.6	(4.3–7.2)	9.2	(7.4–11.4)	9.84	(8.1–11.9)
Average	1.6	(1.0–2.7)	1.8	(1.1–2.8)	6.5	(5.0–8.3)	9.9	(7.8–12.5)	12.5	(10.3–15.1)
Richer	2.2	(1.4–3.4)	2.5	(1.8–3.6)	5.8	(4.4–7.5)	14.4	(11.8–17.3)	16.8	(14.0–20.0)
Richest	4.3	(3.2–5.7)	7.8	(6.0–10.1)	14.2	(11.9–16.8)	23.2	(20.4–26.2)	28.3	(24.5–32.5)
**ANC attendance, visits**										
1–3	1.9	(1.5–2.4)	2.5	(2.0–3.0)	7.2	(6.3–8.2)	11.0	(9.8–12.3)	13.6	(12.1–15.3)
≥4	4.9	(3.3–7.4)	6.8	(5.2–9.0)	10.4	(8.9–12.1)	15.5	(13.8–17.4)	19.6	(17.8–21.5)
Missing	2.1	(1.6–2.8)	2.6	(2.0–3.4)	4.9	(4.0–6.0)	12.2	(10.5–14.2)	12.2	(10.5–14.1)
**Parity**										
1	4.2	(3.0–6.1)	6.0	(4.6–7.9)	15.1	(13.1–17.4)	17.9	(15.7–20.4)	20.7	(18.4–23.1)
2–4	2.6	(2.0–3.3)	3.2	(2.5–4.1)	7.2	(6.1–8.5)	13.8	(12.2–15.5)	16.6	(14.9–18.5)
≥5	1.1	(0.7–1.6)	1.6	(1.2–2.3)	2.8	(2.2–3.7)	6.0	(4.6–7.7)	7.9	(6.4–9.9)
**Baby’s sex**										
Male	2.4	(1.9–3.0)	3.2	(2.6–3.9)	7.9	(7.0–9.0)	14.2	(12.7–15.7)	16.2	(14.7–17.8)
Female	1.9	(1.4–2.6)	2.5	(2.0–3.2)	6.3	(5.5–7.2)	11.4	(10.0–12.9)	14.1	(12.4–16.0)
**Baby’s birth weight**										
Average	6.7	(5.3–8.4)	7.7	(6.3–9.4)	9.0	(7.9–10.2)	12.5	(11.3–13.8)	15.4	(14.0–16.9)
Low birth weight	8.8	(5.0–15.0)	9.5	(5.9–15.0)	10.0	(7.7–12.8)	15.2	(12.0–19.0)	14.7	(12.0–17.8)
Big baby	0.7	(0.5–1.0)	1.1	(0.8–1.5)	4.1	(3.3–5.1)	12.5	(10.3–15.3)	14.3	(11.4–17.7)
**Twin status**										
Singleton	2.0	(1.6–2.4)	2.8	(2.4–3.4)	6.8	(6.1–7.5)	12.0	(11.0–13.2)	14.8	(13.6–16.2)
Multiple	8.6	(3.8–18.4)	4.4	(1.9–10.1)	19.1	(12.3–28.4)	37.2	(27.3–48.4)	27.2	(18.4–38.3)
**Access to information**										
No	0.9	(0.6–1.4)	1.6	(1.0–2.6)	6.4	(4.6–8.9)	9.9	(7.8–12.5)	11.7	(9.6–14.1)
Yes	2.9	(2.3–3.6)	3.2	(2.7–3.9)	7.2	(6.4–8.0)	13.4	(12.1–14.7)	16.1	(14.6–17.7)
**Partner’s education**										
No formal	1.5	(1.0–2.3)	1.3	(0.8–1.9)	5.7	(4.3–7.4)	9.0	(6.8–11.9)	8.9	(6.5–12.1)
Primary	1.9	(1.5–2.6)	2.9	(2.2–3.8)	6.0	(5.2–7.0)	11.8	(10.5–13.1)	13.5	(12.0–15.1)
Secondary and higher	4.4	(2.7–6.9)	5.4	(4.0–7.1)	14.3	(11.3–18.0)	23.6	(20.1–27.7)	25.3	(21.6–29.3)
**Region**										
Kigali City	8.8	(6.8–11.5)	12.1	(8.2–17.5)	15.2	(11.9–19.1)	21.8	(18.0–26.0)	26.4	(22.3–31.0)
South	2.0	(1.3–3.1)	2.8	(2.1–3.8)	7.5	(6.0–9.3)	14.5	(12.1–17.2)	16.2	(13.8–19.0)
West	1.9	(1.3–2.8)	2.8	(2.0–3.9)	6.1	(5.0–7.5)	11.4	(9.2–14.1)	12.5	(10.4–14.9)
East	2.3	(1.4–3.5)	1.9	(1.2–2.9)	6.5	(5.2–8.0)	10.8	(9.0–13.0)	11.2	(8.67–14.4)
North	1.3	(0.8–2.3)	1.2	(0.7–2.1)	4.5	(3.1–6.5)	8.5	(6.4–11.1)	13.2	(10.7–16.3)
**Type of facility**										
Public	8.2	(6.8–10.0)	10.2	(8.6–11.9)	9.6	(8.7–10.7)	13.9	(12.6–15.2)	15.4	(14.1–16.8)
Private	13.6	(7.4–23.8)	10.9	(5.7–20.0)	41.5	(26.6–58.1)	76.5	(36.9–94.8)	60.6	(47.2–72.5)

*ANC* Antenatal care

We found regional disparities in the prevalence of CS across the years. Kigali city had a significantly higher prevalence than the other regions; with a three times increase in CS from 8.8% in 2000 to 26.4% in 2019–20. The Northern region had the lowest prevalence of CS despite a 10 times increase between 2000 (1.3%) and 2019–20 (13.2%) (Table 2).

There was an upward trend in the prevalence of CS in both public and private health facilities. The prevalence of CS in public facilities increased by about seven percentage points from 8.2% (95% CI 6.8–10.0) in 2000 to 15.4% (95% CI 14.1–16.8) in 2019–20. On the other hand, the rate of CS use was about four times high in private facilities from 13.6% (95% CI 7.4–23.8%) in 2000 to 60.6% (95% CI 47.2–72.5) in 2019–20; though the 2019–20 rate was a 20% decrease from the 2014–15 rate (Table 2). The prevalence of CS between public and private health facilities in 2010, 2014–15 and 2019–20 differed significantly despite having almost similar rates in 2005 (Table 2).

### Factors associated with CS in Rwanda

Based on the unadjusted logistic regression models, there was an association between CS and residential area, education, employment, wealth, women’s age, access to media, parity, ANC attendance, multiple births, baby’s sex and birth weight (Suppl. Table 2).

Table 3 presents the results of the population-based multivariable logistic regression models for each of the four surveys. Overall, there were varied associations between CS and maternal age, occupation, wealth, ANC attendance, parity, sex and size of the child, and region of residence over the years. Across the surveys, women with multiple pregnancies, with ≥4 ANC visits, and from the richest households had higher odds of CS, while multiparous women and women with female babies had lower odds of CS. Women with female babies had 19–27% lower odds of CS compared to male babies between 2005 and 2019–20 while those with 5 or more children has 65–85% lower odds of CS compared to those with one child between 2010 and 2019–20. Women with multiple pregnancies had 3.2 to 6.5 times higher odds of CS than singleton pregnancy between 2005 and 2019–20.

**Table 3:** Factors associated with caesarean section at a population level in Rwanda

Characteristics	***2000***	2005	2010	2014	***2019–20***
	aOR (95% CI)	aOR (95% CI)	aOR (95% CI)	aOR (95% CI)	aOR (95% CI)
**Age, years**					
15–19	1	1	1	1	1
20–34	0.76 (0.32–1.78)	1.05 (0.42–2.60)	1.18 (0.67–2.05)	1.12 (0.72–1.72)	1.17 (0.60–2.25)
35–49	1.13 (0.31–4.07)	0.92 (0.33–2.51)	1.86 (0.91–3.79)	1.45 (0.86–2.45)	1.67 (0.82–3.37)
**Residence**					
Urban	1	1	1	1	1
Rural	1.03 (0.45–2.35)	1.07 (0.59–1.95)	0.61 (0.34–1.09)	0.86 (0.59–1.24)	0.80 (0.58–1.12)
**Marital status**†					
Not in a union	1	1	1	1	
Married/cohabiting	1.08 (0.48–2.43)	0.70 (0.37–1.35)	1.97 (1.23–3.12)**	1.01 (0.73–1.40)	
**Education**					
No formal	1	1	1	1	1
Primary	1.66 (0.82–3.34)	0.63 (0.39–1.00)	1.05 (0.72–1.53)	1.27 (0.88–1.83)	1.14 (0.75–1.73)
Secondary	2.68 (0.62–4.57)	1.04 (0.57–1.91)	1.83 (1.06–3.18)*	1.30 (0.83–2.05)	1.26 (0.76–2.08)
**Occupation**					
Not working	1	1	1	1	1
Agriculture	0.27 (0.10–0.72)**	0.76 (0.49–1.17)	0.93 (0.62–1.38)	1.02 (0.69–1.52)	1.00 (0.74–1.35)
Formal employment	0.39 (0.11–1.41)	0.72 (0.42–1.25)	1.34 (0.89–2.01)	1.92 (1.34–2.77)***	1.00 (0 .75–1.32)
**Wealth, quintiles**					
Poorest	1	1	1	1	1
Poorer	0.88 (0.34–2.29)	1.77 (0.84–3.72)	1.02 (0.69–1.52)	0.90 (0.63–1.30)	0.92 (0.64–1.32)
Average	1.06 (0.38–2.99)	1.35 (0.61–3.00)	1.23 (0.84–1.80)	1.02 (0.70–1.49)	1.18 (0.82–1.18)
Richer	1.28 (0.46–3.56)	1.65 (0.79–3.43)	1.02 (0.68–1.52)	1.51 (1.06–2.16)*	1.50 (1.03–2.21)**
Richest	1.69 (0.68–4.21)	2.78 (1.19–6.50)*	1.44 (0.87–2.39)	1.76 (1.15–2.71)*	2.43 (1.58–3.72)**
**ANC attendance, visits**					
1–3	1	1	1	1	1
≥4	1.19 (0.65–2.16)	1.86 (1.22–2.83)**	1.18 (0.93–1.52)	1.27 (1.04–1.57)*	1.44 (1.18–1.75)*
Missing	0.89 (0.59–1.34)	1.16 (0.88–1.53)	0.82 (0.67–1.01)	1.12 (0.93–1.34)	0.91 (0.76–1.09)
**Parity**					
1	1	1	1	1	1
2–4	0.45 (0.25–0.84)*	0.83 (0.49–1.39)	0.48 (0.37–0.64)***	0.64 (0.50–0.81)***	0.84 (0.64–1.11)
5+	0.22 (0.08–0.61)**	0.53 (0.27–1.01)	0.15 (0.10–0.23)***	0.22 (0.14–0.34)***	0.35 (0.23–0.54)**
**Baby’s sex**					
Male	1	1	1	1	1
Female	0.80 (0.55–1.18)	0.73 (0.55–0.97)*	0.81 (0.67–0.97)*	0.79 (0.67–0.94)**	0.81 (0.68–0.96)*
**Baby’s birth weight**					
Average	1	1	1	1	1
Low birth weight	1.49 (0.68–3.27)	1.31 (0.70–2.45)	0.78 (0.53–1.14)	1.06 (0.77–1.44)	0.99 (0.73–1.34)
Big baby	0.14 (0.07–0.26)***	0.20 (0.13–0.32)***	0.59 (0.45–0.78)	1.23 (0.96–1.58)	1.31 (0.97–1.77)
**Twin status**					
Singleton	1	1	1	1	1
Multiple	8.55 (3.36–21.76)***	1.57 (0.57–4.32)	6.11 (3.27–11.40)***	6.49 (3.93–10.74) ***	3.24 (1.84–5.70)**
**Access to information**					
No	1	1	1	1	1
Yes	1.45 (0.70–2.99)	1.27 (0.71–2.28)	0.75 (0.48–1.17)	0.98 (0.70–1.38)	0.92 (0.68–1.24)
**Partner’s education**					
No formal	1	1	1	1	1
Primary	0.60 (0.32–1.13)	1.79 (1.07–2.99)*	0.78 (0.55–1.10)	1.01 (0.74–1.36)	1.23 (0.85–1.80)
Secondary and higher	0.47 (0.21–1.06)	1.03 (0.55–1.95)	0.90 (0.53–1.54)	1.15 (0.77–1.71)	1.40 (0.87–2.25)
**Region**					
Kigali City	1	1	1	1	1
South	0.61 (0.25–1.49)	0.46 (0.23–0.92)*	1.10 (0.51–2.39)	1.10 (0.70–1.72)	1.01 (0.69–1.49)
West	0.84 (0.38–1.89)	0.56 (0.26–1.18)	0.97 (0.43–2.19)	0.98 (0.65–1.48)	0.73 (0.49–1.07)
East	0.74 (0.31–1.73)	0.36 (0.16–0.80)*	1.04 (0.46–2.32)	0.90 (0.59–1.38)	0.74 (0.50–1.10)
North	0.45 (0.17–1.19)	0.22 (0.10–0.51)***	0.74 (0.31–1.75)	0.66 (0.42–1.05)	0.62 (0.40–0.96)*

*p < 0.05; **p < 0.01; ***p < 0.001; *ANC* antenatal care, *CI* confidence interval; † Marital status was excluded in 2019–20 due to multicollinearity

In 2000, women working in the agricultural sector had lower odds of CS (aOR: 0.27, 95% CI 0.10–0.72), while in 2014, women in formal employment had higher odds of CS (aOR: 1.92, 95% CI 1.34–2.77). Partner education and marital status were associated with higher odds of CS in 2005 and 2010, respectively. In 2019–20, highest wealth quintiles, attendance of four or more ANC visits (aOR: 1.44, 95% CI 1.18–1.75), and multiple pregnancies (aOR: 3.24, 95% CI 1.84–5.70) were associated with higher odds of CS while residence in the North region (aOR: 0.62, 95% CI 0.40–0.96), having 5 or more children (aOR: 0.35, 95% CI 0.23–0.54) and female babies (aOR: 0.81, 95% CI 0.68–0.96) were associated with higher odds of CS (Table 3).

The results from the hospital-based model revealed a similar direction of association as those of the population-based model except for higher odds of CS among women who delivered big babies in 2000 (aOR: 2.00, 95% CI 1.11–3.61), 2014–15 (aOR: 1.57, 95% CI 1.22–2.03), 2019–20 and (aOR: 1.84, 95% CI 1.34–1.52) (Table 4).

**Table 4:** Factors associated with caesarean section at the health facility level in Rwanda

Characteristics	***2000***	2005	2010	2014	***2019–20***
	aOR (95% CI)	aOR (95% CI)	aOR (95% CI)	aOR (95% CI)	aOR (95% CI)
**Age, years**					
15–19	1	1	1	1	1
20–34	0.69 (0.29–1.64)	1.26 (0.54–2.92)	1.23 (0.70–2.15)	1.09 (0.71–1.70)	1.18 (0.61–2.25)
35–49	1.06 (0.26–4.22)	1.08 (0.41–2.83)	1.97 (0.95–4.09)	1.43 (0.84–2.42)	1.72 (0.86–3.45)
**Marital status**†					
Not in a union	1	1	1	1	
Married/cohabiting	1.18 (0.51–2.76)	0.63 (0.33–1.23)	1.87 (1.16–3.00)*	1.03 (0.74–1.42)	
**Access to information**					
No	1	1	1	1	1
Yes	1.31 (0.62–2.79)	1.29 (0.69–2.41)	0.73 (0.47–1.14)	0.95 (0.68–1.34)	0.93 (0.69–1.26)
**Education**					
No formal	1	1	1	1	1
Primary	1.68 (0.78–3.63)	0.56 (0.34–0.90)*	1.00 (0.69–1.46)	1.21 (0.84–1.75)	1.06 (0.69–1.63)
Secondary	1.68 (0.56–5.06)	0.89 (0.49–1.61)	1.69 (0.98–2.92)	1.26 (0.81–1.96)	1.18 (0.71–1.97)
**Occupation**					
Not working	1	1	1	1	1
Agriculture	0.31 (0.11–0.92)*	0.83 (0.53–1.30)	0.91 (0.61–1.36)	1.04 (0.70–1.55)	1.02 (0.76–1.37)
Formal employment	0.42 (0.12–1.46)	0.76 (0.45–1.29)	1.24 (0.82–1.87)	1.93 (1.34–2.79)***	1.01 (0.76–1.33)
**Wealth, quintiles**					
Poorest	1	1	1	1	1
Poorer	0.92 (0.32–2.60)	1.57 (0.71–3.45)	1.05 (0.70–1.57)	0.84 (0.58–1.20)	0.86 (0.60–1.23)
Average	0.89 (0.31–2.57)	1.22 (0.53–2.80)	1.21 (0.82–1.78)	0.95 (0.65–1.39)	1.07 (0.74–1.53)
Richer	1.32 (0.47–3.73)	1.27 (0.62–2.62)	0.98 (0.65–1.48)	1.37 (0.96–1.97)	1.31 (0.89–1.91)
Richest	1.56 (0.64–3.81)	1.82 (0.77–4.29)	1.27 (0.77–2.10)	1.57 (1.02–2.43)*	2.10 (1.38–3.19)**
**ANC attendance, visits**					
1–3	1	1	1	1	1
≥4	1.11 (0.61–2.03)	1.56 (1.02–2.39)*	1.09 (0.85–1.39)	1.22 (0.99–1.50)	1.38 (1.13–1.68)*
Missing	0.85 (0.56–1.30)	1.07 (0.80–1.44)	0.86 (0.70–1.06)	1.08 (0.90–1.30)	0.88 (0.73–1.06)
**Parity**					
1	1	1	1	1	1
2–4	0.53 (0.29–0.97)*	1.17 (0.70–1.96)	0.53 (0.40–0.70)***	0.65 (0.51–0.82)***	0.86 (0.66–1.12)
5+	0.29 (0.10–0.86)*	0.87 (0.45–1.68)	0.19 (0.13–0.29)***	0.25 (0.16–0.38)***	0.38 (0.25–0.58)**
**Baby’s sex**					
Male	1	1	1	1	1
Female	0.83 (0.55–1.24)	0.82 (0.61–1.10)	0.85 (0.70–1.03)	0.82 (0.69–0.97)*	0.82 (0.69–0.97)*
**Baby’s birth weight**					
Average	1	1	1	1	1
Low birth weight	1.28 (0.56–2.91)	1.34 (0.73–2.46)	0.80 (0.55–1.16)	1.03 (0.75–1.41)	0.98 (0.72–1.33)
Big baby	2.00 (1.11–3.61)*	1.42 (0.96–2.10)	1.30 (0.99–1.71)	1.57 (1.22–2.03)***	1.84 (1.34–1.52)**
**Twin status**					
Singleton	1	1	1	1	1
Multiple	11.07 (3.36–36.5) ***	1.32 (0.50–3.50)	6.08 (3.20–11.56)***	6.43 (3.88–10.66)***	3.63 (2.04–6.48)***
**Region**					
Kigali City	1	1	1	1	1
South	0.52 (0.21–1.29)	0.38 (0.18–0.78)**	1.04 (0.49–2.20)	1.09 (0.70–1.69)	1.00 (0.68–1.47)
West	0.75 (0.33–1.71)	0.53 (0.26–1.10)	0.85 (0.39–1.86)	0.94 (0.62–1.41)	0.71 (0.48–1.04)
North	0.38 (0.13–1.12)	0.19 (0.08–0.44)***	0.70 (0.30–1.62)	0.64 (0.41–1.02)	0.61 (0.39–0.94)*
East	0.70 (0.30–1.63)	0.35 (0.16–0.78)**	0.92 (0.42–2.00)	0.88 (0.57–1.34)	0.72 (0.49–1.06)
**Residence**					
Urban	1	1	1	1	1
Rural	1.24 (0.63–2.42)	1.19 (0.67–2.14)	0.61 (0.34–1.10)	0.87 (0.60–1.26)	0.82 (0.59–1.14)
**Partners education**					
No formal	1	1	1	1	1
Primary	0.46 (0.23–0.92)*	1.62 (0.95–2.75)	0.73 (0.52–1.03)	0.97 (0.72–1.31)	1.23 (0.84–1.80)
Secondary and higher	0.35 (0.14–0.86)*	0.83 (0.44–1.55)	0.85 (0.50–1.45)	1.09 (0.74–1.61)	1.40 (0.86–2.25)

*p < 0.05; **p < 0.01; ***p < 0.001; † Marital status was excluded in 2019–20 due to multicollinearity

## Discussion

Over the past two decades, the rate of CS in Rwanda significantly increased from 2.2 to 15.6%, with disproportionately high rates among high socioeconomic groups and in urban areas, private health facilities and Kigali City. The rising trend in CS in Rwanda mirrors the global trend which shows an average annual increase of 4% [8]. The current CS rate of 15.6% in Rwanda is three times the rate in SSA, [10] and more than twice the average rate in Eastern and Southern African [38] though a previous study in Rwanda has shown a higher CS rate of 21% [39]. The increasing CS rate in Rwanda could indicate increased access to EmONC services and the high utilization of skilled delivery [40]. The current rate of CS is slightly above the 10–15% WHO threshold for beneficial CS and likely to continue increasing if measures are not taken. Importantly, despite a 20% decrease in the prevalence of CS in private hospitals between 2014 and 2019–20, there is a need to continue to monitor CS in private health facilities where the rate was four times higher compared to public health facilities in 2019–20.

Overall, women of high socioeconomic status (education, occupation, and wealth) had increased odds of CS delivery, which reflects findings from previous studies [11, 41–44]. CS is a high-cost procedure in most settings making it accessible mostly to the rich. However, in Rwanda, a majority of women are covered under the community-based health insurance schemes that have increased access to affordable healthcare by minimising the out-of-pocket expenditure and reducing the socioeconomic disparities in access and use of CS [20]. This is further evidenced by the increased proportion of institutional deliveries from 27%^26^ in 2000 to 28% [24], 69% [25], 91% [22] and 93% [21] in the year 2005, 2010, 2015 and 2019–20 respectively. Moreover, women with high education levels are associated with ANC attendance [43, 45, 46], providing them with an opportunity to receive health education and counselling and early detection of risks. Mothers with high-risk pregnancies are also more likely to utilise ANC services than those with normal pregnancies though ANC attendance could also be indicative of better access to healthcare including CS. Sayinzoga et al. further attributed improved maternal health services including access to CS use to established network of follow up for pregnant women, infrastructural development and proper leadership in health service delivery [47]. This could explain why about 75% of the districts in Rwanda provided CS services in 2016.

Multiple pregnancies are high-risk and one of the obstetric indications for CS [48, 49]. In our study, women with multiple pregnancies had higher odds of CS similar to previous studies [41, 50, 51]. In 2014, the rate of CS among women with multiple pregnancies was three times the average rate of CS in Rwanda, having doubled within the preceding five years. However, in 2019–20, the rate of CS among women with multiple pregnancies reduced by 10 percentage points while that of singleton significantly increased to 23.3%. Hence, there is a need to review the indication for CS among women with multiple and singleton pregnancies to guide clinicians on women who are likely to benefit from the procedure and avoid unnecessary CS in this group.

Our study found lower odds of CS delivery among women who gave birth to female babies compared to those who delivered male babies similar to previous studies [52–57]. Male babies tend to be heavier than female babies [58–60] and are at an increased risk of adverse pregnancy outcomes [58]. We also found that women with two or more children had lower odds of CS delivery compared to primiparous women. This finding is consistent with previous studies in Kenya [61] and Ethiopia [51] and could be possibly attributed to poor dissolution of cervical collagen fibre in primigravid women due to their inexperience in labour [62]. Also, women who may have delivered their first baby via CS may have had successful vaginal delivery after a previous CS [63].

Big babies in the population-based analyses had lower odds of CS but had higher odds of CS in the hospital-based analyses compared to normal-weight babies. Hospital-based studies have found a similar association between CS and big babies [64]. Large babies present difficulty in delivery possibly due to insufficient passage and prolonged labour. The lower odds of CS among big babies in the population-based analyses could be attributed to a delay in weighing of the babies delivered at home, who may have gained weight by the time of presenting at a health facility but also the fact that having a big baby is not an indication for CS and thus most big babies are delivered vaginally.

Our study adds to the evidence on CS in Rwanda by examining the population- and hospital-based trends and factors associated with CS in Rwanda over the past two decades using repeated nationally representative data making the findings generalisable to Rwanda. Our study, however, has some limitations that should be considered when interpreting the findings. First, there is a potential for overestimation or underestimation due to self-reporting of most variables and that the variables were measured at the time of the survey rather than at the time of delivery. Second, the dataset had missing observations and the study only included variables available from the dataset and could have missed important factors that predict CS in Rwanda. Finally, we could not assess the differences between the private and public health facilities due to the small sample of women who delivered in private health facilities.

## Conclusion

Over the past 20 years, the rate of CS in Rwanda has seen an upward trend increasing seven times between 2000 and 2019–20 at health facility and population-level with persistent regional disparities over the years. The rates and odds of CS have been disproportionately high among women of high socioeconomic groups, and those who resided in urban areas and Kigali City, used private health facilities, had multiple pregnancies, and attended at least four antenatal care visits. Women in Rwanda seem to have increased access to and use of CS. However, the significant increase in the rate of CS is of concern due to the potential of unnecessary CS. Therefore, there is a need to examine the guidelines for CS use in Rwanda to ensure proper indications for use of CS are adopted for beneficial outcomes.

## Supplementary Information

**Additional file 1: Supplementary Figure 1**: Flowchart of the study sample.

**Additional file 2: Supplementary Table 1**. Operational definition of the study variables.

**Additional file 3: Supplementary Table 2**. Factors associated with caesarean section at population level.

**Additional file 4: Supplementary Table 3**. STROBE Statement—Checklist of items that should be included in reports of cross-sectional studies.

## Data Availability

The Rwanda Demographic Health Survey data used in this study is available on the Demographic Health Survey website at www.dhsprogram.com on request.

## Abbreviations

ANC: Antenatal Care
AOR: Adjusted Odds Ratio
BMI: Body Mass Index
CI: Confidence Interval
COR: Crude Odds Ratio
CS: Caesarean Section
DHS: Demographic Health Surveys
SSA: Sub-Saharan Africa
SDGs: Sustainable Development Goals
WHO: World Health Organization
