# Supplementary figures and images for: Prevalence and factors associated with caesarean section in Rwanda: a trend analysis of Rwanda demographic and health survey 2000 to 2019–20
Source: BMC Pregnancy Childbirth. 2022 May 16;22:410. doi: 10.1186/s12884-022-04679-y (PMC9112592; doi:10.1186/s12884-022-04679-y)

**
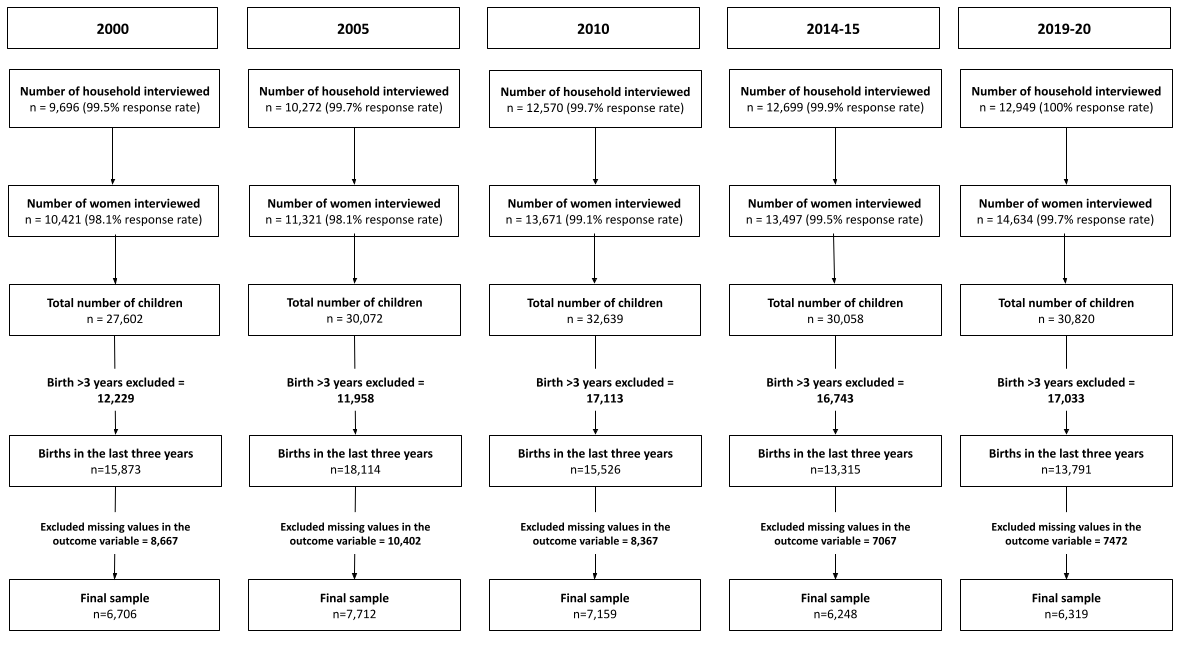
**

Supplement: Supplementary file 1 — Additional file 1: Supplementary Figure 1: Flowchart of the study sample. [file 12884_2022_4679_MOESM1_ESM.docx]
