# Supplementary material for: Prevalence and factors associated with caesarean section in Rwanda: a trend analysis of Rwanda demographic and health survey 2000 to 2019–20
Source: BMC Pregnancy Childbirth. 2022 May 16;22:410. doi: 10.1186/s12884-022-04679-y (PMC9112592; doi:10.1186/s12884-022-04679-y)
Supplement: Supplementary file 2 — Additional file 2: Supplementary Table 1. Operational definition of the study variables. [file 12884_2022_4679_MOESM2_ESM.docx]

|  | **Study variables** | **Operational definition** |
| --- | --- | --- |
| **Outcome variable** | Caesarean section | The dependent variable is CS delivery among currently married women aged 15–49 for their most recent birth in the three years before the survey. The variable used the responses to one question in the RDHS: “Was (NAME) delivered by caesarean, that is, did they cut your belly open to take the baby out?” with a binary outcome of “yes” or “no” categories. |
| **Explanatory variables** |  |  |
| **Sociodemographic** | Maternal age | Age of mother in years at the time of the interview was categorized into four categories: 15–19, 20–34 and 35–49 years |
|  | Marital status | Mother’s marital status at the time of the interview. Categorized into not–in–a–union and in–a–union (married /cohabiting) |
|  | Access to information | Composite variable based on whether they have watched, read, or listened to television, newspapers, and radio over the past week respectively (Yes or No) |
| **Socioeconomic** | Education level | Mother’s level of education at the time of the interview (Secondary & higher, primary, no formal education) |
|  | Occupation | Mother occupation at the time of the interview was categorised as not working, agriculture/self–employed and formal employment |
|  | Wealth index | Wealth index was developed using principal component analysis of household assets ((drinking water, source of non–drinking water, toilet facility, electricity, television, refrigerator, bicycle, motorcycle, car, floor material, wall material, roof material) (Poorest, Poorer, Middle, Richer, Richest). |
| **Obstetrics** | Antenatal care visits | Number of times ANC visits during pregnancy (<4 times, ≥4 times, Missing) |
|  | Parity | Number of previous births (1, 2–4, 5+) |
| **Community** | Provinces | Mother’s province of residence at the time of the interview as per the administrative levels within the country (Kigali city, North, West, East and South) |
|  | Residence | Rural or urban |
| **Child-related** | Sex | Sex of the children; male or female |
|  | Twin status | Child’s twin status (singleton, multiple) |
|  | Birth weight | Low birth weight (<2500g), average (2500–4000 g) and big baby (>4000g) |
| **Partner–related** | Partner's education level | Partner’s level of education at the time of the interview (Secondary & higher, primary, no formal education) |
