# Supplementary material for: Prevalence and factors associated with caesarean section in Rwanda: a trend analysis of Rwanda demographic and health survey 2000 to 2019–20
Source: BMC Pregnancy Childbirth. 2022 May 16;22:410. doi: 10.1186/s12884-022-04679-y (PMC9112592; doi:10.1186/s12884-022-04679-y)
Supplement: Supplementary file 3 — Additional file 3: Supplementary Table 2. Factors associated with caesarean section at population level. [file 12884_2022_4679_MOESM3_ESM.docx]

| **Characteristics** | ***2000*** | **2005** | **2010** | **2014** | ***2019–20*** |
| --- | --- | --- | --- | --- | --- |
|  | **cOR (95% CI)** | **cOR (95% CI)** | **cOR (95% CI)** | **cOR (95% CI)** | **cOR (95% CI)** |
| **Age, years** |  |  |  |  |  |
| 15–19 | 1 | 1 | 1 | 1 | 1 |
| 20–34 | 0.59 (0.30–1.15) | 0.74 (0.40–1.39) | 0.72 (0.49–1.07) | 0.94 (0.66–1.37) | 1.13 (0.76–1.68) |
| 35–49 | 0.40 (0.17–0.94)* | 0.42 (0.20–0.87)* | 0.50 (0.31–0.80)** | 0.65 (0.43–1.00)* | 0.94 (0.94–1.46) |
| **Marital status** |  |  |  |  |  |
| Not in a union | 1 | 1 | 1 | 1 | 1 |
| Married/cohabiting | 1.39 (0.80–2.41) | 0.69 (0.44–1.10) | 0.89 (0.67–1.19) | 0.97 (0.77–1.22) | 1.05 (0.85–1.29) |
| **Access to information** |  |  |  |  |  |
| No | 1 | 1 | 1 | 1 | 1 |
| Yes | 3.21 (1.99–5.18)*** | 2.03 (1.20–3.43)** | 1.12 (0.78–1.60) | 1.40 (1.07–1.84)* | 1.45 (1.13–1.86)** |
| **Education** |  |  |  |  |  |
| No formal | 1 | 1 | 1 | 1 | 1 |
| Primary | 2.21 (1.28–3.84)** | 1.12 (0.73–1.71) | 1.26 (0.91–1.74) | 1.65 (1.18–2.32)** | 1.49(1.07–2.07)* |
| Secondary | 5.02 (2.80–9.00) | 4.09 (2.60–6.46)*** | 4.21 (2.82–6.29) | 3.47 (2.42–4.99)*** | 3.28(2.24–4.80)*** |
| **Occupation** |  |  |  |  |  |
| Not working | 1 | 1 | 1 | 1 | 1 |
| Agriculture | 0.17 (0.06–0.43)*** | 0.40 (0.27–0.59)*** | 0.59 (0.44–0.79)*** | 0.69 (0.52–0.91)** | 0.59(0.46–0.75)*** |
| Formal employment | 0.78 (0.27–2.29) | 1.60 (1.04–2.46)* | 1.59 (1.09–2.34)* | 2.01 (1.46–2.76)*** | 0.82(0.67–1.01) |
| **Wealth, quintiles** |  |  |  |  |  |
| Poorest | 1 | 1 | 1 | 1 | 1 |
| Poorer | 0.63 (0.29–1.38) | 2.01 (0.99–4.10) | 1.01 (0.72–1.42) | 0.95 (0.69–1.30) | 0.94(0.69–1.27) |
| Average | 1.01 (0.45–2.30) | 1.75 (0.84–3.63) | 1.18 (0.84–1.64) | 1.02 (0.73–1.44) | 1.23(0.91–1.67) |
| Richer | 1.35 (0.67–2.70) | 2.49 (1.30–4.77)** | 1.04 (0.75–1.46) | 1.56 (1.14–2.14)** | 1.74(1.28–2.35)*** |
| Richest | 2.72 (1.42–5.22) | 8.07 (4.31–15.12)*** | 2.82 (2.11–3.77)*** | 2.81 (2.15–3.68)*** | 3.40(2.55–4.52)*** |
| **ANC attendance, visits** |  |  |  |  |  |
| 1–3 | 1 | 1 | 1 | 1 | 1 |
| ≥4 | 2.71 (1.68–4.35)*** | 2.89 (2.02–4.14)*** | 1.48 (1.19–1.86)*** | 1.49 (1.24–1.78)*** | 1.54(1.30–1.83)*** |
| Missing | 1.11 (0.81–1.52) | 1.05 (0.80–1.38) | 0.66 (0.54–0.80)*** | 1.12 (0.94–1.34) | 0.88(0.75–1.04) |
| **Parity** |  |  |  |  |  |
| 1 | 1 | 1 | 1 | 1 | 1 |
| 2–4 | 0.60 (0.39–0.91)* | 0.51 (0.34–0.75)*** | 0.43 (0.34–0.56)*** | 0.73 (0.60–0.90)** | 0.76(0.64–0.91)** |
| 5+ | 0.24 (0.14–0.42)*** | 0.26 (0.17–0.40)*** | 0.16 (0.12–0.22)*** | 0.29 (0.21–0.40)*** | 0.33(0.25–0.44)*** |
| **Baby’s sex** |  |  |  |  |  |
| Male | 1 | 1 | 1 | 1 | 1 |
| Female | 0.78 (0.55–1.12) | 0.79 (0.62–1.01) | 0.78 (0.66–0.92)** | 0.78 (0.66–0.91)** | 0.85(0.72–1.00)* |
| **Baby’s birth weight** |  |  |  |  |  |
| Average | 1 | 1 | 1 | 1 | 1 |
| Low birth weight | 1.34 (0.72–2.51) | 1.26 (0.72–2.19) | 1.13 (0.83–1.53) | 1.25 (0.94–1.65) | 0.95(0.74–1.21) |
| Big baby | 0.09 (0.06–0.14)*** | 0.14 (0.10–0.19) | 0.43 (0.33–0.56)*** | 1.00 (0.80–1.26) | 0.91(0.71–1.19) |
| **Twin status** |  |  |  |  |  |
| Singleton | 1 | 1 | 1 | 1 | 1 |
| Multiple | 4.63 (1.88–11.5)*** | 1.61 (0.66–3.94) | 3.25 (1.92–5.47) | 4.34 (2.74–6.86)*** | 2.15(1.31–3.54)** |
| **Region** |  |  |  |  |  |
| Kigali City | 1 | 1 | 1 | 1 | 1 |
| South | 0.21 (0.12–0.36)*** | 0.21 (0.12–0.36)*** | 0.45 (0.31–0.65)*** | 0.61 (0.44–0.83)** | 0.54 (0.40–0.73)*** |
| West | 0.20 (0.12–0.33)*** | 0.21 (0.12–0.36)*** | 0.36 (0.26–0.52)*** | 0.46 (0.33–0.65)*** | 0.40 (0.29–0.54)*** |
| North | 0.14 (0.07–0.26)*** | 0.09 (0.05–0.18)*** | 0.27 (0.17–0.42)*** | 0.33 (0.23–0.49)*** | 0.35 (0.24–0.51)*** |
| East | 0.24 (0.14–0.41)*** | 0.14 (0.08–0.26)*** | 0.39 (0.27–0.55)*** | 0.44 (0.32–0.60)*** | 0.42 (0.30–0.59)*** |
| **Residence** |  |  |  |  |  |
| Urban | 1 | 1 | 1 | 1 | 1 |
| Rural | 0.21 (0.14–0.31) | 0.27 (0.19–0.40)*** | 0.34 (0.25–0.44) | 0.43 (0.34–0.54)*** | 0.41(0.33–0.52)*** |
| **Partners education** |  |  |  |  |  |
| No formal | 1 | 1 | 1 | 1 | 1 |
| Primary | 1.28 (0.77–2.15) | 2.36 (1.44–3.86)*** | 1.07 (0.78–1.46) | 1.35 (0.99–1.83) | 1.59 (1.12–2.27)* |
| Secondary and higher | 2.95 (1.57–5.56) | 4.46 (2.62–7.61)*** | 2.79 (1.89–4.11) | 3.13 (2.16–4.53)*** | 3.45(2.35–5.07)*** |
